# Molecular basis of a new ovine model for human 3M syndrome-2

**DOI:** 10.1186/s12863-020-00913-8

**Published:** 2020-09-15

**Authors:** S. A. Woolley, S. E. Hayes, M. R. Shariflou, F. W. Nicholas, C. E. Willet, B. A. O’Rourke, I. Tammen

**Affiliations:** 1 Faculty of Science, Sydney School of Veterinary Science, The University of Sydney, Camden, NSW 2570 Australia; 2 Sydney Informatics Hub, Core Research Facilities, The University of Sydney, Sydney, NSW 2006 Australia; 3 NSW Department of Primary Industries, Elizabeth Macarthur Agricultural Institute, Menangle, NSW 2568 Australia

**Keywords:** Whole genome sequencing, Sheep, Inherited disease, Lethal, Frameshift variant, Animal model, 3M syndrome-2

## Abstract

**Background:**

Brachygnathia, cardiomegaly and renal hypoplasia syndrome (BCRHS, OMIA 001595–9940) is a previously reported recessively inherited disorder in Australian Poll Merino/Merino sheep. Affected lambs are stillborn with various congenital defects as reflected in the name of the disease, as well as short stature, a short and broad cranium, a small thoracic cavity, thin ribs and brachysternum. The BCRHS phenotype shows similarity to certain human short stature syndromes, in particular the human 3M syndrome-2. Here we report the identification of a likely disease-causing variant and propose an ovine model for human 3M syndrome-2.

**Results:**

Eight positional candidate genes were identified among the 39 genes in the approximately 1 Mb interval to which the disease was mapped previously. Obscurin like cytoskeletal adaptor 1 (*OBSL1*) was selected as a strong positional candidate gene based on gene function and the resulting phenotypes observed in humans with mutations in this gene. Whole genome sequencing of an affected lamb (BCRHS3) identified a likely causal variant ENSOARG00000020239:g.220472248delC within *OBSL1*. Sanger sequencing of seven affected, six obligate carrier, two phenotypically unaffected animals from the original flock and one unrelated control animal validated the variant. A genotyping assay was developed to genotype 583 animals from the original flock, giving an estimated allele frequency of 5%.

**Conclusions:**

The identification of a likely disease-causing variant resulting in a frameshift (p.(Val573Trpfs*119)) in the OBSL1 protein has enabled improved breeding management of the implicated flock. The opportunity for an ovine model for human 3M syndrome and ensuing therapeutic research is promising given the availability of carrier ram semen for BCRHS.

## Background

**Fig. 1:**
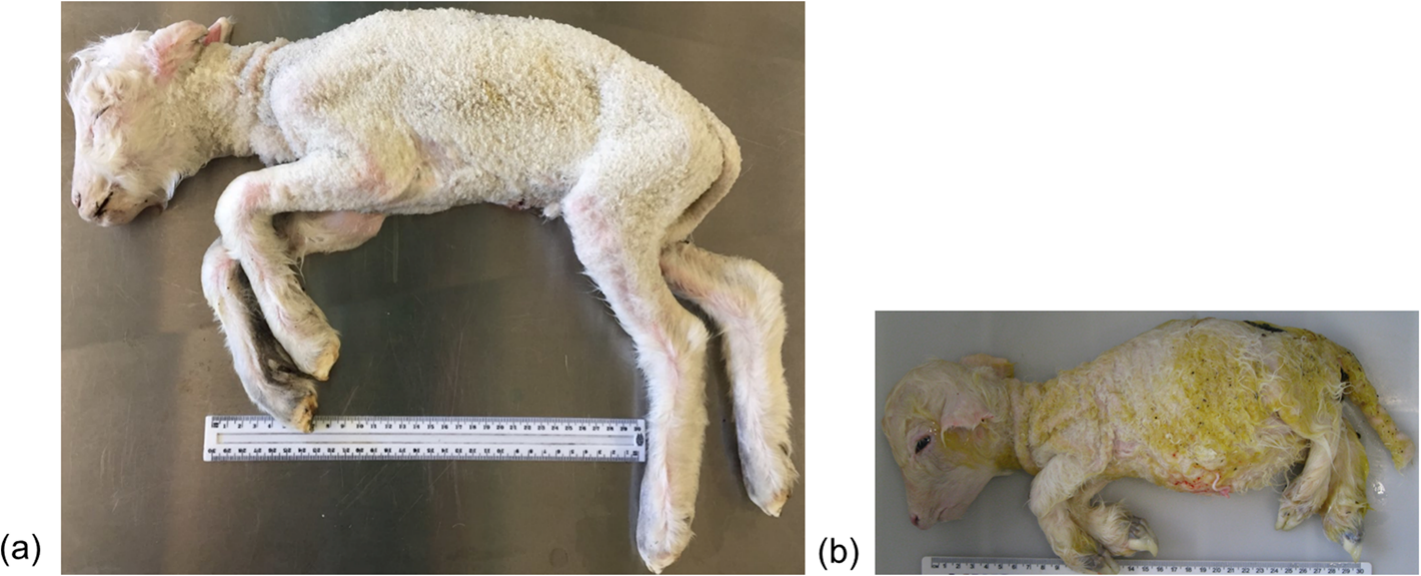
Comparison between an unrelated control stillborn Merino lamb **a** and a BCRHS-affected stillborn lamb **b**. The image of the affected lamb highlights some of the characteristic clinical signs: short stature, shortened broad cranium, brachygnathia and small thoracic cavity (Ruler length in both images = 30 cm)

Brachygnathia, cardiomegaly and renal hypoplasia syndrome (BCRHS, OMIA 001595–9940) is a previously reported lethal inherited disorder in Australian Poll Merino/Merino sheep [1, 2] that, to the best of our knowledge, has not been reported in other sheep breeds in Australia. This disorder is characterized by a range of congenital defects and conforms with an autosomal recessive mode of inheritance based on previous pedigree information and segregation analyses [1]. Affected lambs are stillborn and the primary defects associated with this disorder are brachygnathia, cardiomegaly and renal hypoplasia, with additional skeletal defects including short stature, a short and broad cranium, a small thoracic cavity reduced in size by approximately 25%, thin ribs and brachysternum (Fig. 1). Affected lambs also present with congestive hepatopathy and small kidneys, which are reduced in size by approximately 50%, with male affected lambs having bilateral cryptorchidism [1]. Overall, these findings suggest a syndromic growth disorder in affected lambs.

Growth disorders leading to short stature in humans can be broadly grouped into two main categories: disproportionate short stature where height and some body proportions are reduced in length; and proportionate short stature, where overall height is reduced but all body proportions remain within normal limits [3]. Short stature or dwarfism in livestock can arise from selective breeding for small body phenotypes [4]. Short stature phenotypes can also arise in livestock as an undesired trait, with underlying complex or Mendelian inheritance [5].

Multiple cases of inherited short stature in non-human species have been listed in Mouse Genome Informatics (MGI) and Online Mendelian Inheritance in Animals (OMIA) databases. Inherited forms are common in mice [6] and many genes have been implicated in cattle, sheep, goats, horses, pigs, rabbits, dogs, cats, chickens, Japanese quail and Sumatran tigers [7] (Additional file 1: Table S1). The BCRHS condition with its multitude of defects, in addition to dwarfism, represents an opportunity to further elucidate human disorders with similar phenotypes. A number of inherited short stature syndromes in humans have been reported, including Seckel, Mulibrey Nanism, Bloom, Meier–Gorlin, microcephalic osteodysplastic primordial dwarfism types I and II, Silver-Russell and 3M syndromes [8, 9]. Overlapping clinical signs are observed between BCRHS and a number of these syndromes, with particular similarity observed with the phenotypes of Silver-Russel and 3M syndromes [9–11]. Human 3M syndrome has been subcategorized into three types, 3M1 (OMIM 273750) with causal mutations located in the Cullin 7 (*CUL7*) gene, 3M2 (OMIM 612921) with causal mutations located in the Obscurin like cytoskeletal adaptor 1 (*OBSL1*) gene, and 3M3 (OMIM 614205 [12];) with causal mutations located in the Coiled-Coil Domain Containing 8 (*CCD8)* gene. The phenotypic similarity between BCRHS and certain human short stature syndromes, in particular 3M syndrome-2, adds further potential for developing a large animal model for the disease.

Shariflou et al. (2012) mapped BCRHS to a 1.1 megabase (Mb) region on ovine chromosome OAR2, flanked by single nucleotide polymorphisms (SNP) s50915 and s40177, using a medium density ovine SNP chip for genotyping, followed by genome-wide association and homozygosity analyses. At that time, 25 genes were predicted to be located in the 1.1 Mb segment [2], as only an early virtual ovine genome assembly was available, based on a comparative mapping approach that mapped sheep DNA segments onto bovine and other mammalian genomes [13]. Since the publication of the Shariflou et al. (2012) study, the *Ovis aries* Oar_v3.1 genome assembly (GCA_000298735.1) became available. Therefore, the aim of the present study was to identify the gene and causal mutation for BCRHS by analyzing the region flanked by SNPs s50915 and s40177 from whole genome sequence data aligned to the Oar_v3.1 reference genome.

## Results

### Identification of positional candidate genes

Thirty-nine genes were identified in the region flanked by SNPs s50915 and s40177 identified by Shariflou et al. (2012), which corresponds to position OAR2:g.220083076–221052836 on the Oar_v3.1 genome assembly (GCA_000298735.1). Twenty-five of these genes were identified to code for known proteins, six genes coded for uncharacterized proteins and eight were RNA genes (Additional file 2: Table S2). Eight protein coding genes were identified as functional positional candidates for BCRHS (Additional file 3: Table S3), and were prioritized based on known function and the extent to which causal mutations in these genes produced phenotypes similar to the BCRHS phenotype. The *OBSL1* gene was selected as the strongest candidate based on this approach.

### Whole genome sequencing

Whole genome sequencing of an affected lamb (BCRHS3) identified 11,671 raw variants in an interval that included the region of interest plus an additional 1 Mb flanking sequence (OAR2:219083025–222052887) on the Oar_v3.1 genome assembly (GCA_000298735.1). After filtering and removal of known Single Nucleotide Polymorphism Database (dbSNP) variants, 103 variants with a predicted ‘low’, ‘moderate’ or ‘high’ impact on protein function that were homozygous alternate in BCRHS3 and not homozygous alternate in the control Merino sheep Y0346 were identified (Additional file 4: Table S4). Twenty-six of the 103 variants were located within three of the top eight prioritized positional candidate genes. Fifteen variants were located in *OBSL1*, 10 variants in the chondroitin polymerizing factor (*CHPF*) gene and one variant in the GDP-mannose pyrophosphorylase A (*GMPAA*) gene (Additional file 4: Table S4). Visual inspection of these 26 variants using SAMtools tview [14] in BCRHS3 and three control genomes (Merino sheep Y0346 and Y0244 and one Persian sheep), revealed that all variants for *CHPF* and *GMPAA* were present in these unrelated control animals and were therefore unlikely to be disease-causing. Seven variants located within the *CHPF* gene located at positions OAR2:220443181, 220443186, 220443189, 220443192, 220443194, 220443200 and 220443202 were in the same region of poor sequencing quality across all controls and the affected BCRHS animal, and were therefore not further considered.

**Fig. 2:**
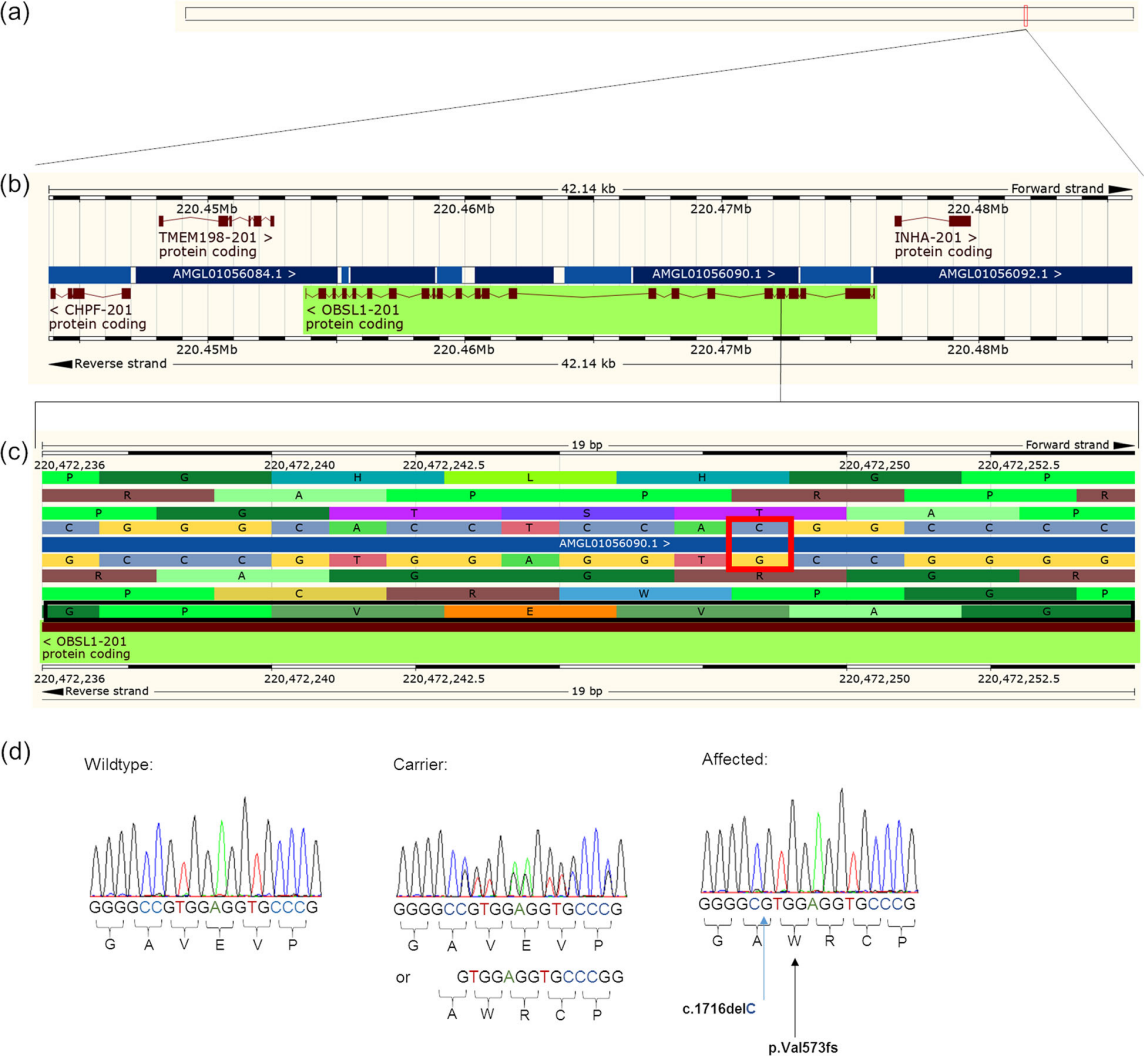
Schematic diagram of the ovine *OBSL1* gene showing the location of the candidate causal mutation ENSOARG00000020239:g.220472248delC with Sanger sequencing chromatograms for one wildtype control, one obligate carrier and one affected animal. **a** Location of the ovine *OBSL1* gene, OAR2:220453801–220475937 on the Oar_v3.1 ovine genome assembly. **b** Enlarged view of the *OBSL1* gene with 25 exons. **c** Genomic region containing the c.1716delC frameshift variant with protein translation frames obtained from Ensembl genome browser 98 (Ensembl, accessed 26th December 2019, < http://asia.ensembl.org/Ovis_aries/Location/View?db=core;g=ENSOARG00000020239;r=2:220472236-220472254;t=ENSOART00000022037>. The position of the variant is identified by a red box and the protein reading frame is identified by a black box. **d** Sanger sequencing chromatograms for one wildtype, one obligate carrier and one affected animal (reverse strand)

**Fig. 3:**
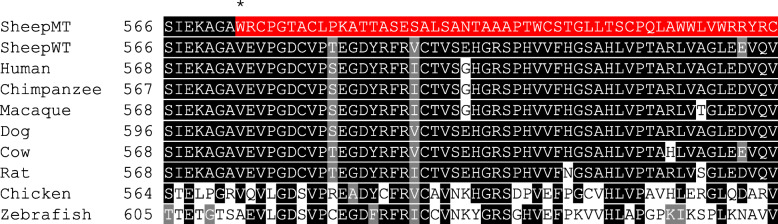
Multiple species partial OBSL1 protein alignment using T-Coffee and BOXSHADE. Accessions used were XP_027821027.1 (*Ovis aries*), NP_056126.1 (*Homo sapiens*), XP_003309516.1 (*Pan troglodytes*), XP_002799127.1 (*Macaca mulatta*), XP_005640767.1 (*Canis lupus familiaris*), NP_001068959.2 (*Bos taurus*), XP_343600.4 (*Rattus norvegicus*), XP_003641689.2 (*Gallus gallus*) and NP_001121829.1 (*Danio rerio*). SheepMT refers to the mutant *Ovis aries* sequence and SheepWT refers to the wildtype *Ovis aries* sequence. The predicted frameshift starting with the replacement of valine with tryptophan in position 573 in affected sheep is highlighted by an asterix (*), with the partial new amino acid sequence highlighted in red in the SheepMT sequence, that is predicted to terminate at positon 691

Fourteen of the *OBSL1* variants were observed in controls or in areas of very low sequencing coverage, leaving only one strong candidate variant. This variant was a single nucleotide deletion ENSOARG00000020239:g.220472248delC; ENSOART00000022037.1:c.1716delC (Fig. 2; XM_027965226.1:g.236304071delC or XM_027965226.1:c.1716delC on the new Oar_rambouillet_v1 genome assembly (GCA_002742125.1)). This results in a frameshift of the OBSL1 protein after the valine amino acid at residue position 573 (p.(Val573Trpfs*119); Fig. 3) and a prematurely truncated protein. The National Center for Biotechnology Information Open Reading Frame (NCBI ORF) Finder [15] predicted the ovine OBSL1 wildtype sequence start codon to begin at nucleotide 55 and the stop codon to end at nucleotide 5754, with an amino acid length of 1899. The mutant sequence containing the c.1716delC variant results in a frameshift with the last nucleotide of the stop codon at nucleotide 2130, yielding a predicted altered amino acid length of 691 (p.(Val573Trpfs*119)) and a 64% truncation of OBSL1 (Additional file 6: Fig. S1). The c.1716delC variant is predicted to alter the amino acid sequence (Additional file 7) within a conserved fibronectin type 3 domain resulting in the truncation of this domain as well as the loss of four immunoglobulin (Ig) domains (Additional file 8: Fig. S2).

### Validation of c.1716delC

**Fig. 4:**
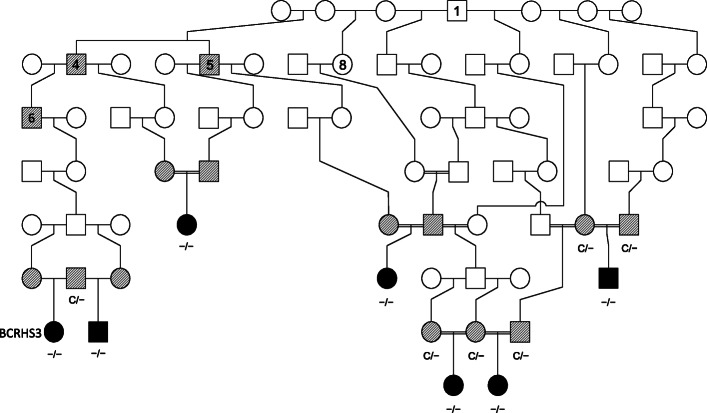
Partial pedigree showing segregation of the c.1716delC mutation (C = wildtype, − = deletion) with the BCRHS phenotype. The pedigree links animals in this study to three obligate carriers (animals 4, 5 and 6) and two suspected carriers (animals 1 and 8) identified in a detailed pedigree by Shariflou et al. (2011). Females and males are denoted by circles and squares, respectively. Filled symbols represent affected animals and shaded symbols represent obligate carriers. The affected animal whole genome sequenced in this study is indicated by ‘BCRHS3’

Segregation of the c.1716delC variant with BCRHS was initially investigated in seven affected (including BCRHS3), six obligate carrier and two phenotypically unaffected animals from the same flock, and one control Merino sheep from an unrelated flock. Polymerase chain reaction (PCR) products were amplified for all 16 samples and Sanger sequenced (Table 1). The results supported segregation of the c.1716delC variant with BCRHS (Fig. 4). All seven affected animals were homozygous for the deletion, six obligate carrier animals were heterozygous and two phenotypically unaffected and one unrelated control animals were homozygous wildtype (Fig. 2).

**Table 1:** Sanger sequencing genotype results for the ENSOARG00000020239:g.220472248delC variant identified within the *OBSL1* gene

Sample ID	Phenotype	Reference	Alternate	Genotype of individual
Control_1	Unaffected	CC	C-	CC/CC
Control_2	Unaffected	CC	C-	CC/CC
Control_3	Unaffected	CC	C-	CC/CC
Carrier_1	Obligate carrier	CC	C-	CC/C-
Carrier_2	Obligate carrier	CC	C-	CC/C-
Carrier_3	Obligate carrier	CC	C-	CC/C-
Carrier_4	Obligate carrier	CC	C-	CC/C-
Carrier_5	Obligate carrier	CC	C-	CC/C-
Carrier_6	Obligate carrier	CC	C-	CC/C-
BCRHS3	Affected	CC	C-	C-/C-
BCRHS11	Affected	CC	C-	C-/C-
Affected_3	Affected	CC	C-	C-/C-
Affected_4	Affected	CC	C-	C-/C-
Affected_5	Affected	CC	C-	C-/C-
Affected_6	Affected	CC	C-	C-/C-
Affected_7	Affected	CC	C-	C-/C-

The c.1716delC variant was not listed as a known variant in the Ensembl Genome Browser and was not present in the variant database from 935 sequenced sheep processed by Agriculture Victoria Research staff.

### TaqMan PCR genotyping assay

A custom TaqMan PCR genotyping assay (Additional file 9: Fig. S3) was developed to genotype an additional 583 animals from the current cohort of sheep in the original flock, revealing 61 heterozygous animals and 522 homozygous wildtype animals, giving an estimated allele frequency of 5%.

## Discussion

Our study identified a novel likely causal variant, c.1716delC, in the ovine *OBSL1* gene in a lamb affected with BCRHS by analyzing whole genome sequence within a genomic region previously associated with the disease. This variant was validated in additional animals and the development of a discriminatory genotyping assay has facilitated improved breeding management practices. Moreover, screening of the original flock has revealed a high estimated minor allele frequency of 5%. These results are a continuation of the work reported by Shariflou et al. (2011; 2012). These studies first described the clinical signs and pathology of BCRHS, and after extensive pedigree analyses, identified this disorder as a recessively inherited Mendelian trait that was mapped to a 1.1 Mb region on OAR2.

Inherited growth disorders are relatively common, with numerous genes identified for these disorders in both humans and livestock [3, 16]. Short stature syndromes such as 3M syndrome, have been characterized in humans and are a part of a group of clinically heterogeneous growth disorders.

While eight genes were initially considered as positional candidate genes, the identification of 26 of 103 private variants within only three of these genes - *OBSL1*, *CHPF* and *GMPAA* - allowed for further prioritization of candidate genes based on gene function and mutation association with disease. Of all the positional candidate genes within the identified region, *OBSL1* was considered the strongest candidate as mutations in this gene cause 3M syndrome-2 in humans (OMIM 612921) [12]. This autosomal recessive growth disorder results in growth, facial and skeletal abnormalities in pre- and postnatal children with similar phenotypes to BCRHS cases [17, 18].

The *OBSL1* gene encodes a cytoskeletal adaptor protein that is involved in cell interactions and the cell matrix [19]. Mutations within *OBSL1* have been associated with human 3M syndrome-2, a short stature growth disorder [20] that shows similar clinical signs to BCRHS-affected lambs. The clinical signs in humans resulting from mutations in the remaining two positional candidate genes, *CHPF* and *GMPAA*, did not show the same degree of similarity with the clinical signs of BCRHS-affected lambs [21–23]. The *CHPF* gene is involved in cell division and cytokinesis, with defects within this gene resulting in defective early embryogenesis through the arrestment of cell division [21]. Mutations within the *GMPAA* gene have been reported to be involved with defects associated with neurological impairment and facial abnormalities [22, 23]. Whole genome sequencing provided further support for the selection of *OBSL1* as the prime candidate gene. Of the 26 variants that passed filtering located within the top three positional candidate genes, only the c.1716delC *OBSL1* variant was predicted to impact protein function and observed in the homozygous non-reference state in the single affected animal.

The c.1716delC variant results in a frameshift after the valine amino acid residue at position 573 in the OBSL1 protein, where the remaining amino acid sequence is completely changed thereafter (Fig. 3, Additional file 6). Four ovine OBSL1 isoforms have been predicted, each with differing amino acid residue lengths of 1899 amino acids in OBSL1 isoform X1 (XP_027821027), 1807 amino acids in isoform X2 (XP_027821028), 1802 amino acids in isoform X3 (XP_027821029) and 1023 amino acids in isoform X4 (XP_027821030). Similarly, in humans, three different OBSL1 isoforms exist with a 1896 amino acid sequence length for the OBSL1 isoform 1 precursor (NP_056126), 1543 amino acids for the isoform 2 precursor (NP_001166902) and 1025 amino acids for the isoform 3 precursor (NP_001166879). The truncation of the ovine OBSL1 isoform X1 from the c.1716elC variant imparts a 64% loss of the protein, resulting in a shortened amino acid length from 1899 to 691 amino acid residues (Fig. 4). The early termination of the protein and the ensuing loss of four conserved Ig domains (Additional file 8: Fig. S2) indicates that protein function may be diminished. The Ig domains located within the OBSL1 protein play important roles in forming interactive binding sites for other proteins and forming complexes with muscle proteins such as titin [24, 25]. The OBSL1 protein has been shown to form a direct complex with CUL7, named the cullin complex, through binding at the CUL7 C-terminus [25, 26]. It is therefore plausible that due to the p.(Val573Trpfs*119) and the ensuing truncation and loss of four Ig domains, the binding ability and interaction of the OBSL1 protein in the cullin complex could be altered through loss of available protein binding sites. Further protein interaction and modelling studies are required to fully understand the binding behavior of the mutated ovine OBSL1 in BCRHS-affected sheep, and its interaction with the CUL7 protein.

The *OBSL1* gene was considered as a strong candidate, based on the phenotypic similarity between 3M syndrome-2 in humans and the BCRHS-affected lambs, as well as its biological function as a widespread cytoskeletal adaptor protein important for tissue stabilization in multiple organs [19]. In brief, clinical signs of 3M syndrome-2 include a large head, frontal bossing, short nose and triangular-shaped face with a pointed chin during later years in life, a short neck and thorax, thin ribs, slender long bones and tall vertebral bodies [17, 18]. Endocrine function and growth hormone levels are within normal limits for affected children [17, 27]. The presentation of BCRHS-affected lambs showcasing multiple congenital defects including brachygnathia, short stature, a short and broad cranium, a small thoracic cavity, thin ribs and brachysternum [1] draw obvious similarities to the clinical signs displayed by children affected by 3M syndrome-2. However, BCRHS-affected lambs also present with congestive hepatopathy and small kidneys [1].

A majority of disease-causing mutations for human 3M syndrome are located within one of three genes, with approximately 70% of cases occurring within the *CUL7* gene, 25% in the *OBSL1* gene and 5% within the *CCD8* gene [28, 29]. It is important to note that there have been no observed phenotypic differences between human patients with either *CUL7* or *OBSL1* mutations [20]. Mutations within the human *OBSL1* gene for 3M syndrome-2 typically occur within the first six to eight exons [17, 20] and affect all three human isoforms. The c.1716delC variant identified in this study is similarly located within the sixth exon of the ovine *OBSL1* gene (Fig. 2). Hanson et al. (2009) used a gene knockout model to show that OBSL1 appears to play a role in regulating CUL7 protein levels in cells and therefore, may act in a common pathway. Interaction studies conducted by Hanson et al. (2011) showed that the OBSL1 protein acts as an adaptor protein for CUL7 and CCD8, despite the lack of interaction between the CUL7 and CCD8 proteins [30].

To determine the protein effect of the c.1716delC variant for BCRHS-affected sheep, similar gene knockout models and protein interaction assays would be beneficial to further elucidate the impact of this variant on disease phenotype. As no affected animals are currently available for further study, CRISPR-Cas9 [31] could be utilized to replicate this variant to introduce an ovine cell culture gene knockout model. Protein levels of OBSL1, CUL7 and CCD8 could be investigated to confirm whether this common pathway also exists within sheep, and whether altering this pathway and complex through OBSL1 truncation results in the BCRHS phenotype.

Silver-Russel syndrome is recognized as a differential diagnosis for all three subcategories of 3M syndrome, as it is characterized by slow growth before and after birth [17]. Skeletal surveys and radiology are often used to help differentiate between these two possible diagnoses, as Silver-Russel syndrome patients do not display the skeletal phenotypes observed in 3M syndrome patients [32]. Treatment of human 3M syndrome often involves growth hormone administration, however the efficiency of this treatment has not been determined [17].

The use of animal models to assist in advancing the knowledge of human disease has been proven to be beneficial [33]. The sheep investigated within this study would be prime candidates for an ovine model of human 3M syndrome-2. The affected sheep appear to suffer from a more severe phenotype compared to humans with 3M syndrome-2, and the development of a large animal model would enable further disease characterization on both the molecular and protein level to evaluate therapeutic interventions by using a model with comparable organ size scaling [34].

## Conclusions

The c.1716delC variant described in this study results in a frameshift mutation and the premature truncation of the OBSL1 protein. Variant segregation among our ovine study set and similarity of the BCRHS-affected ovine phenotype to human 3M syndrome-2 suggests that BCRHS-affected sheep represent an ovine model for human 3M syndrome-2. The discovery of this variant has enabled the development of a robust genotyping assay, that is being used for the identification of carrier animals and for improved breeding management. The availability of a large animal model for human 3M syndrome-2 represents a unique opportunity to further investigate the biochemical basis of human 3M syndrome-2 as well as offering alternatives to validate therapeutic interventions in preclinical trials.

## Methods

### Animals and DNA isolation

Brachygnathia, cardiomegaly with renal hypoplasia was reported from a single Merino/Poll Merino flock in Australia and samples were collected by the owner. Tissue samples (liver, kidney, heart and ear notches) were collected from stillborn lambs or slaughter animals and either stored in RNA*later* or frozen. Blood cards were collected as per diagnostic DNA testing protocols [35].

Genomic DNA for whole genome sequencing was extracted from tissue stored in RNA*later* (ThermoFisher Scientific, DE, USA) from two affected animals. Genomic DNA for Sanger sequencing was extracted from frozen tissue for an additional five affected lambs, six obligate carriers and two unaffected Merino sheep from the same flock. All extractions used the QIAGEN DNeasy Blood & Tissue Kit following the manufacturer’s Animal Tissues Spin-Column protocol (QIAGEN, CA, USA).

Genomic DNA for the genotyping assay was isolated from blood cards collected from 583 Merino sheep from the original flock using a standard blood card digest protocol [36]. DNA was also available from one additional Merino sheep from an unrelated flock.

Pedigree information was compiled from over 40 years of breeding records, with details presented in Shariflou et al. (2011). Pedigraph [37] and the R package kinship2 version 1.8.4 [38] were used for drawing pedigree trees.

### Identification of positional candidate genes

The flanking SNPs s50915 and s40177 for the 1.1 Mb region reported by Shariflou et al. (2012) were used to identify the region of interest in the Ensembl Oar_v3.1 genome assembly (GCA_000298735.1), where the region is smaller in size at approximately 0.97 Mb (OAR2(CM001583.1):g.220083076–221052836). Genes in this region were identified using the University of California Santa Cruz (UCSC) Genome Browser (http://genome.ucsc.edu/) and Table Browser and Ensembl annotations [39–41]. Online databases Online Mendelian Inheritance in Man (OMIM) [12], MGI [6] and PubMed were used to identify positional candidate genes based on the normal function of the protein or any reported phenotypes that were similar to BCRHS (Additional file 5: Table S5).

### Whole genome sequencing

Genomic DNA concentration and purity of samples from two affected lambs (BCRHS3 and BCRHS11) were measured using the NanoDrop 8000 spectrophotometer and Qubit® 3.0 fluorometer (Thermo Scientific, DE, USA) and visualized on a 1% agarose gel.

Due to financial constraints as well as quantity and quality measures, only BCRHS3 was submitted for whole genome sequencing. Three additional sheep whole genome sequenced for the investigation of other unrelated inherited conditions (Merino sheep Y0244 and Y0346 and one Persian sheep, all from different flocks) were used as controls in the present study. Whole genome sequencing was performed using the Illumina HiSeq™ X Ten sequencing platform (Illumina, San Diego, CA) by the Kinghorn Centre for Clinical Genomics (Garvan Institute of Medical Research, Darlinghurst, Australia). DNA libraries were prepared using the Illumina® TruSeq DNA Nano Library Prep kit. Each sample was sequenced as 150 base pair (bp) paired-end reads at an expected 30X coverage. Adaptor sequences were removed by the service provider. Quality visualization and control was conducted on the resulting sequence reads using FastQC (version 0.11.3) (https://www.bioinformatics.babraham.ac.uk/projects/fastqc/). Inspection of FastQC output indicated that the sequence data for all four samples were of good quality (yield ranged from 54.87 Gb to 80.16 Gb, 76.15 to 97.42% > PHRED30, 40.5 to 42.5% GC content and no adaptor contamination flagged). Therefore no quality trimming was conducted.

### Read mapping, variant calling and annotation

Paired-end sequence reads were mapped to the *Ovis aries* Oar_v3.1 genome assembly (GCA_000298735.1) using Burrows-Wheeler Aligner (BWA-mem) version 0.7.15 [42] with default settings. Polymerase chain reaction (PCR) duplicates were marked using samblaster version 0.1.22 [43]. Lane-level binary alignment maps (BAMs) were merged using Picard version 1.119 (http://broadinstitute.github.io/picard/). Sorting and indexing was performed with SAMtools [14] version 1.6. Local realignment around insertion and deletion sites as well as base quality score recalibration using known variants downloaded from Ensembl’s dbSNP database for *Ovis aries* version 87 [41] were performed with the Genome Analysis Toolkit (GATK) version 3.7.0 [44, 45].

Single nucleotide polymorphisms were called using GATK HaplotypeCaller in GVCF mode [46] and were genotyped using GATK GenotypeGVCFs [44, 45]. Annotation and prediction of functional effects of SNP on OAR2 was conducted using SnpEff [47] version 4.3 and the Ensembl annotation release 86 for Oar_v3.1.

### Variant filtering

Variants annotated by SnpEff [47] within the 0.97 Mb region of interest and an additional 1 Mb flanking on OAR2 were selected for filtering using a case-control approach in SnpSift [48] version 4. Variants that were homozygous alternate for BCRHS3 and that were not homozygous alternate for control Merino sheep Y0346 were selected. Variants were filtered for ‘low’, ‘moderate’ or ‘high’ impact on protein function as annotated by SnpEff [47], with known dbSNP variants and duplicate variants manually removed.

Of these, variants present in the prioritized positional candidate genes underwent visual inspection using SAMtools tview in the sequence data for BCRHS3, Y0346, an additional Merino (Y0244) and one Persian sheep. This reduced the list of candidate variants by excluding SNPs in regions of poor sequencing quality or those that were present in the controls.

### Validation of c.1716delC

Following PCR amplification of the region flanking the c.1716delC variant, Sanger sequencing was conducted to validate the variant in seven affected, six obligate and three unaffected control animals (Table 1).

PrimerBLAST [49] was used to design a primer pair to amplify the region flanking the ENSOART00000022037.1:c.1716delC (Oar_v3.1) variant in the candidate gene *OBSL1*. PCR amplification of a 229 bp product was performed using a Gradient Palm-Cycler™ Thermal Cycler (CGI-96, Corbett Life Science, NSW, Australia) in a total volume of 25 μL, containing 1x Platinum™ SuperFi™ PCR Master Mix (Invitrogen, ThermoFisher Scientific, DE, USA), 0.5 μM of each primer F2 5′- GTGTTGGCCGAAATGTTCAAG-3′ and R2 5′-GTTCGCTGACAGTGCAGACTC-3 and approximately 50 ng of genomic DNA. The initial denaturation step was performed at 98 °C for 30 s, followed by 35 cycles consisting of a denaturation step at 98 °C for 10 s, annealing at 64 °C for 10 s and extension at 72 °C for 30 s. A final extension was performed at 72 °C for 5 min. PCR products were visualised on a 2% agarose gel before submission to Macrogen (Seoul, Korea) for DNA sequencing.

Sequencing data was analysed using MEGAX software [50] by aligning the sequences to genomic DNA to identify variants. Variants were compared to the variant database in Ensembl [51] and predicted impacts of novel variants on protein function were additionally determined by SIFT analysis [52]. Cross-species OBSL1 protein alignments were conducted using T-Coffee [53] and BOXSHADE (v3.2) across nine species. These included OBSL1 protein sequences from sheep (*Ovis aries*), human (*Homo sapiens*), chimpanzee (*Pan troglodytes*), Rhesus macaque (*Macaca mulatta*), dog (*Canis lupus familiaris*), cattle (*Bos taurus*), Brown rat (*Rattus norvegicus*), chicken (*Gallus gallus*) and zebrafish (*Danio rerio*).

To assess the predicted impact of the c.1716delC variant on OBSL1 length, NCBI ORF Finder [15] was used to compare the wildtype and mutant mRNA sequences from the ovine OBSL1 isoform X1 (XP_027821027) to identify alternative stop codon sites in the mutant sequence. To identify predicted losses of conserved ovine OBSL1 protein domains, the NCBI Conserved Domains database [15, 54] was used for the mutant OBSL1 mRNA sequence (XM_027965226.1) containing the c.1716delC variant.

To assess whether the c.1716delC variant had been previously reported in sheep, known ovine *OBSL1* variants in the Ensembl genome browser (https://www.ensembl.org/Ovis_aries/Gene/Variation_Gene/Table?db=core;g=ENSOARG00000020239;r=2:220453801-220475937;t=ENSOART00000022037) were investigated.

Presence of the variant was also screened in an additional database of sequence variants generated by the Agriculture Victoria Research team at the Centre For AgriBioscience, Melbourne. These variants were discovered from 935 sheep sequences: 453 from the SheepGenomesDB Project and 482 contributed by the Sheep CRC Project [55]. A range of different breeds were represented including 127 purebred Australian Merino sheep.

### TaqMan PCR genotyping assay

A custom TaqMan real-time PCR assay was designed using the Custom TaqMan® Assay Design tool (ThermoFisher Scientific, DE, USA) to discriminate between homozygous wildtype, heterozygous and homozygous mutant genotypes.

Allelic discrimination was performed using the ViiA™ 7 system (Applied Biosystems™, CA, USA) in a final reaction volume of 12.5 μL. Each reaction contained 1 x TaqMan® Genotyping Master Mix (Applied Biosystems, CA, USA), 900 nmol/L of assay specific primers 5′- CGGTAGGCACGCAGTCC-3′ and 5′-TACAGTGCTTCAGCATTGAGAAAG-3′, 250 nmol/L of allele specific 5′-VIC-CACCTCCACGCCCCG-NFQ-3′ (wildtype) and 5′-FAM-CCTCCACGGCCCCG-NFQ-3′ (mutant) probes and approximately 10–30 ng of genomic DNA. Each assay commenced with a pre-read stage at 60 °C for 30 s followed by an initial denaturation at 95 °C for 10 min, followed by 45 cycles of denaturation at 95 °C for 15 s, annealing and extension at 60 °C for 60 s and a final post-read stage at 60 °C for 30 s. Genotypes were analysed using the QuantStudio™ Real-Time PCR System version 1.3 (Applied Biosystems™, CA, USA).

## Supplementary information

**Additional file 1: Table S1.** Adapted Online Mendelian Inheritance in Animals (OMIA) list of reported inherited forms of short stature (or dwarfism) in cattle, sheep, a goat, horses, pigs, rabbits, dogs, a cat, chickens, Japanese quail and a Sumatran tiger with the associated OMIA ID.

**Additional file 2: Table S2.** List of genes identified in the region flanked by SNPs s50915 and s40177 corresponding to positions OAR2:g.220083076–221052836 on the Oar_v3.1 genome assembly.

**Additional file 3: Table S3.** Top eight protein coding positional candidate genes identified in the OAR2:g.220083076–221052836 region on the Oar_v3.1 genome assembly.

**Additional file 4: Table S4.** List of 103 private whole genome sequencing variants identified in an affected lamb (BCRHS3) after filtering based on segregation, predicted protein impact, removal of known SNPs and duplicates.

**Additional file 5: Table S5.** Gene list obtained from Mouse Genome Informatics (MGI) with PubMed literature counts identifying genes causing similar phenotypes to BCRHS.

**Additional file 6: Figure S1.** Comparison of predicted open reading frames (ORF) for OBSL1 mRNA (XM_027965226.1) and predicted mutant ovine mRNA for OBSL1 using NCBI ORF Finder [15] (accessed 18th December 2019, < https://www.ncbi.nlm.nih.gov/orffinder/>). (a) ORF1 (black *) represents the ORF that codes for OBSL1 (1899 amino acid residues). (b) ORF1 (red *) codes for a truncated and modified protein of 691 amino acid residues.

**Additional file 7.** Ovine OBSL1 isoform X1 protein sequences (XP_027821027) for wildtype (SheepWT) and mutant (SheepMT) sheep. The predicted mutant c.1716delC (p.(Val573Trpfs*119)) altered amino acid sequence is highlighted in red.

**Additional file 8: Figure S2.** Wildtype OBSL1 protein showing conserved domains obtained from the NCBI Conserved Domains database [15, 54] (accessed 18th December 2019, <https://www.ncbi.nlm.nih.gov/Structure/cdd/wrpsb.cgi>). The location of the c.1716delC variant is indicated. The resulting modified protein p.(Val573Trpfs*119) is predicted to have a truncated fibronectin type 3 domain and is lacking four immunoglobulin domains.

**Additional file 9: Figure S3.** Allelic discrimination plot visualised using QuantStudio™ Real-Time PCR System version 1.3 (Applied Biosystems™) for a TaqMan genotyping assay used to discriminate the ENSOART00000022037.1:c.1716delC variant for homozygous wildtype (red dots), heterozygous (green dots), homozygous mutant (blue) individuals and a no DNA template control (black square).

## Data Availability

The dataset generated and/or analysed during the current study are available at the European Nucleotide Archive (www.ebi.ac.uk/ena/) and was deposited under the study accession number PRJEB39179 and the sample accession is SAMEA7034587.

## Abbreviations

BCRHS: Brachygnathia, cardiomegaly and renal hypoplasia syndrome
bp: Base pair
CCD8: Coiled-Coil Domain Containing 8
CHPF: Chondroitin polymerizing factor
CUL7: Cullin 7
dbSNP: Single Nucleotide Polymorphism Database
DNA: Deoxyribonucleic acid
GMPAA: GDP-mannose pyrophosphorylase A
Mb: Megabase
MGI: Mouse Genome Informatics
NCBI ORF: National Center for Biotechnology Information Open Reading Frame Finder
NCBI: National Center for Biotechnology Information
OAR2: *Ovis aries* chromosome 2
OBSL1: Obscurin like cytoskeletal adaptor 1
OMIA: Online Mendelian Inheritance in Animals
OMIM: Online Mendelian Inheritance in Man
PCR: Polymerase chain reaction
SNP: Single nucleotide polymorphism

## References

[1] Shariflou MR, Wade CM, Windsor PA, Tammen I, James JW, Nicholas FW (2011). Lethal genetic disorder in poll merino/merino sheep in Australia. Aus Vet J.

[2] Shariflou MR, Wade CM, Kijas J, McCulloch R, Windsor PA, Tammen I (2012). Brachygnathia, cardiomegaly and renal hypoplasia syndrome (BCRHS) in merino sheep maps to a 1.1-megabase region on ovine chromosome OAR2. Anim Genet.

[3] Boegheim IJM, Leegwater PAJ, van Lith HA, Back W (2017). Current insights into the molecular genetic basis of dwarfism in livestock. Vet J.

[4] Parnell PF, Arthur PF, Barlow R (1997). Direct response to divergent selection for yearling growth rate in Angus cattle. Livest Prod Sci.

[5] Cavanagh J, Tammen I, Windsor P, Bateman J, Savarirayan R, Nicholas F (2007). Bulldog dwarfism in Dexter cattle is caused by mutations in ACAN. Mamm Genome.

[6] Bult CJ, Blake JA, Smith CL, Kadin JA, Richardson JE (2019). Mouse genome database (MGD) 2019. Nucleic Acids Res.

[7] Online Mendelian Inheritance in Animals. Sydney School of Veterinary Science, University of Sydney, Sydney. 2019:https://omia.org/ Accessed 26th December 2019.

[8] Boycott KM, Vanstone MR, Bulman DE, Mackenzie AE (2013). Rare-disease genetics in the era of next-generation sequencing: discovery to translation. Nat Rev Genet.

[9] Clayton PE, Hanson D, Magee L, Murray PG, Saunders E, Abu-Amero SN (2012). Exploring the spectrum of 3-M syndrome, a primordial short stature disorder of disrupted ubiquitination. Clin Endocrinol.

[10] Silver HK, Kiyasu W, George J, Deamer WC (1953). Syndrome of congenital hemihypertrophy, shortness of stature, and elevated urinary gonadotropins. Pediatrics..

[11] Russell A (1954). A syndrome of intra-uterine dwarfism recognizable at birth with cranio-facial dysostosis, disproportionately short arms, and other anomalies (5 examples). Proc Roy Soc Med.

[12] Online Mendelian Inheritance in Man. Johns Hopkins University (Baltimore, MD). 2019. https://omim.org/.Accessed 26th December 2019.

[13] Archibald AL, Cockett NE, Dalrymple BP, Faraut T, Kijas JW, The International Sheep Genomics Consortium (2010). The sheep genome reference sequence: a work in progress. Anim Genet.

[14] Li H, Handsaker B, Wysoker A, Fennell T, Ruan J, Homer N (2009). The sequence alignment/map format and SAMtools. Bioinformatics..

[15] NCBI Resource Coordinators (2018). Database resources of the National Center for biotechnology information. Nucleic Acids Res.

[16] Argente J, Tatton-Brown K, Lehwalder D, Pfäffle R (2019). Genetics of growth disorders—which patients require genetic testing?. Front Endocrinol.

[17] Huber C, Munnich A, Cormier-Daire V (2011). The 3M syndrome. Best Pract Res Clin Endocrinol Metab.

[18] Murray PG, Hanson D, Coulson T, Stevens A, Whatmore A, Poole RL (2013). 3-M syndrome: a growth disorder associated with IGF2 silencing. Endocr Connect.

[19] Geisler SB, Robinson D, Hauringa M, Raeker MO, Borisov AB, Westfall MV (2007). Obscurin-like 1, OBSL1, is a novel cytoskeletal protein related to obscurin. Genomics..

[20] Hanson D, Murray PG, Sud A, Temtamy SA, Aglan M, Superti-Furga A (2009). The primordial growth disorder 3-M syndrome connects Ubiquitination to the cytoskeletal adaptor OBSL1. Am J Hum Genet.

[21] Izumikawa T, Kitagawa H, Mizuguchi S, Nomura KH, Nomura K, Tamura JI (2004). Nematode chondroitin polymerizing factor showing cell−/organ-specific expression is indispensable for chondroitin synthesis and embryonic cell division. J Biol Chem.

[22] Gold WA, Sobreira N, Wiame E, Marbaix A, Van Schaftingen E, Franzka P (2017). A novel mutation in GMPPA in siblings with apparent intellectual disability, epilepsy, dysmorphism, and autonomic dysfunction. Am J Med Genet A.

[23] Koehler K, Malik M, Mahmood S, Gießelmann S, Beetz C, Hennings JC (2013). Mutations in GMPPA cause a glycosylation disorder characterized by intellectual disability and autonomic dysfunction. Am J Hum Genet.

[24] Sauer F, Vahokoski J, Song YH, Wilmanns M (2010). Molecular basis of the head-to-tail assembly of giant muscle proteins obscurin-like 1 and titin. EMBO Rep.

[25] Benian GM, Mayans O (2015). Titin and Obscurin: giants holding hands and discovery of a new Ig domain subset. J Mol Biol.

[26] Litterman N, Ikeuchi Y, Gallardo G, O'Connell BC, Sowa ME, Gygi SP (2011). An OBSL1-Cul7Fbxw8 ubiquitin ligase signaling mechanism regulates Golgi morphology and dendrite patterning. PLoS Biol.

[27] Al-Dosari MS, Al-Shammari M, Shaheen R, Faqeih E, Alghofely MA, Boukai A (2012). 3M syndrome: an easily recognizable yet underdiagnosed cause of proportionate short stature. J Pediatr.

[28] Hanson D, Murray PG, Coulson T, Sud A, Omokanye A, Stratta E (2012). Mutations in CUL7, OBSL1 and CCDC8 in 3-M syndrome lead to disordered growth factor signalling. J Mol Endocrinol.

[29] Huber C, Fradin M, Edouard T, Le Merrer M, Alanay Y, Da Silva DB (2010). OBSL1 mutations in 3-M syndrome are associated with a modulation of IGFBP2 and IGFBP5 expression levels. Hum Mutat.

[30] Hanson D, Murray PG, Sullivan J, Urquhart J, Daly S, Bhaskar SS (2011). Exome sequencing identifies CCDC8 mutations in 3-M syndrome, suggesting that CCDC8 contributes in a pathway with CUL7 and OBSL1 to control human growth. Am J Hum Genet.

[31] Hsu PD, Lander ES, Zhang F (2014). Development and applications of CRISPR-Cas9 for genome engineering. Cell..

[32] Akawi NA, Ali BR, Hamamy H, Al-Hadidy A, Al-Gazali L (2011). Is autosomal recessive Silver-Russel syndrome a separate entity or is it part of the 3-M syndrome spectrum?. Am J Med Genet A.

[33] Robinson NB, Krieger K, Khan FM, Huffman W, Chang M, Naik A (2019). The current state of animal models in research: a review. Int J Surg.

[34] Davidson MK, Lindsey JR, Davis JK (1987). Requirements and selection of an animal model. Isr J Med Sci.

[35] NSW Department of Primary Industries. Sample Collection Guide Blood Cards. 2017:https://www.dpi.nsw.gov.au/__data/assets/pdf_file/0019/701335/sample-collection-guide-blood-card.pdf Accessed 29th June 2020.

[36] O’Rourke BA, Kelly J, Spiers ZB, Shearer PL, Porter NS, Parma P (2017). Ichthyosis fetalis in polled Hereford and shorthorn calves. J Vet Diagn Investig.

[37] Garbe JR, Da Y. Pedigraph: A Software Tool for the Graphing and Analysis of Large Complex Pedigree. https://animal.geneumnedu/pedigraph. User manual Version 24, Department of Animal Science, University of Minnesota (2008) Accessed February 2020.

[38] Sinnwell JP, Therneau TM, Schaid DJ (2014). The kinship2 R package for pedigree data. Hum Hered.

[39] Kent WJ, Sugnet CW, Furey TS, Roskin KM, Pringle TH, Zahler AM (2002). The human genome browser at UCSC. Genome Res.

[40] Karolchik D, Hinrichs AS, Furey TS, Roskin KM, Sugnet CW, Haussler D (2004). The UCSC table browser data retrieval tool. Nucleic Acids Res.

[41] Zerbino DR, Achuthan P, Akanni W, Amode MR, Barrell D, Bhai J (2017). Ensembl 2018. Nucleic Acids Res.

[42] Li H, Durbin R (2009). Fast and accurate short read alignment with burrows-wheeler transform. Bioinformatics..

[43] Faust GG, Hall IM (2014). SAMBLASTER: fast duplicate marking and structural variant read extraction. Bioinformatics..

[44] McKenna A, Hanna M, Banks E, Sivachenko A, Cibulskis K, Kernytsky A (2010). The genome analysis toolkit: a MapReduce framework for analyzing next-generation DNA sequencing data. Genome Res.

[45] DePristo MA, Banks E, Poplin R, Garimella KV, Maguire JR, Hartl C (2011). A framework for variation discovery and genotyping using next-generation DNA sequencing data. Nat Genet.

[46] Van der Auwera GA, Carneiro MO, Hartl C, Poplin R, del Angel G, Levy-Moonshine A (2013). From FastQ data to high confidence variant calls: the Genome Analysis Toolkit best practices pipeline. Curr Protoc Bioinformatics.

[47] Cingolani P, Platts A, Wang Le L, Coon M, Nguyen T, Wang L (2012). A program for annotating and predicting the effects of single nucleotide polymorphisms, SnpEff: SNPs in the genome of Drosophila melanogaster strain w1118; iso-2; iso-3. Fly..

[48] Cingolani P, Patel V, Coon M, Nguyen T, Land S, Ruden D (2012). Using Drosophila melanogaster as a model for Genotoxic chemical mutational studies with a new program. SnpSift Front Genet.

[49] Ye J, Coulouris G, Zaretskaya I, Cutcutache I, Rozen S, Madden T (2012). Primer-BLAST: a tool to design target-specific primers for polymerase chain reaction. BMC Bioinformatics.

[50] Kumar S, Stecher G, Tamura K (2016). MEGA7: molecular evolutionary genetics analysis version 7.0 for bigger datasets. Mol Biol Evol.

[51] McLaren W, Pritchard B, Rios D, Chen Y, Flicek P, Cunningham F (2010). Deriving the consequences of genomic variants with the Ensembl API and SNP effect predictor. Bioinformatics..

[52] Flanagan SE, Patch AM, Ellard S (2010). Using SIFT and PolyPhen to predict loss-of-function and gain-of-function mutations. Genet Test Mol Biomarkers.

[53] Di Tommaso P, Moretti S, Xenarios I, Orobitg M, Montanyola A, Chang J-M, et al.. T-Coffee: A web server for the multiple sequence alignment of protein and RNA sequences using structural information and homology extension. Nucleic Acids Res. 2011;39(Web Server issue):W13-WW7. 10.1093/nar/gkr245 PMC3125728 21558174

[54] Marchler-Bauer A, Bo Y, Han L, He J, Lanczycki CJ, Lu S (2017). CDD/SPARCLE: functional classification of proteins via subfamily domain architectures. Nucleic Acids Res.

[55] Daetwyler HD, Brauning R, Chamberlain AJ, McWilliam S, McCulloch A, Vander Jagt CJ (2017). 1000 Bull Genomes And Sheepgenomedb Projects: Enabling Costeffective Sequence Level Analyses Globally. Proc Assoc Advmt Anim Breed Genet.

