# Supplementary figures and images for: Molecular basis of a new ovine model for human 3M syndrome-2
Source: BMC Genet. 2020 Sep 15;21:106. doi: 10.1186/s12863-020-00913-8 (PMC7493961; doi:10.1186/s12863-020-00913-8)

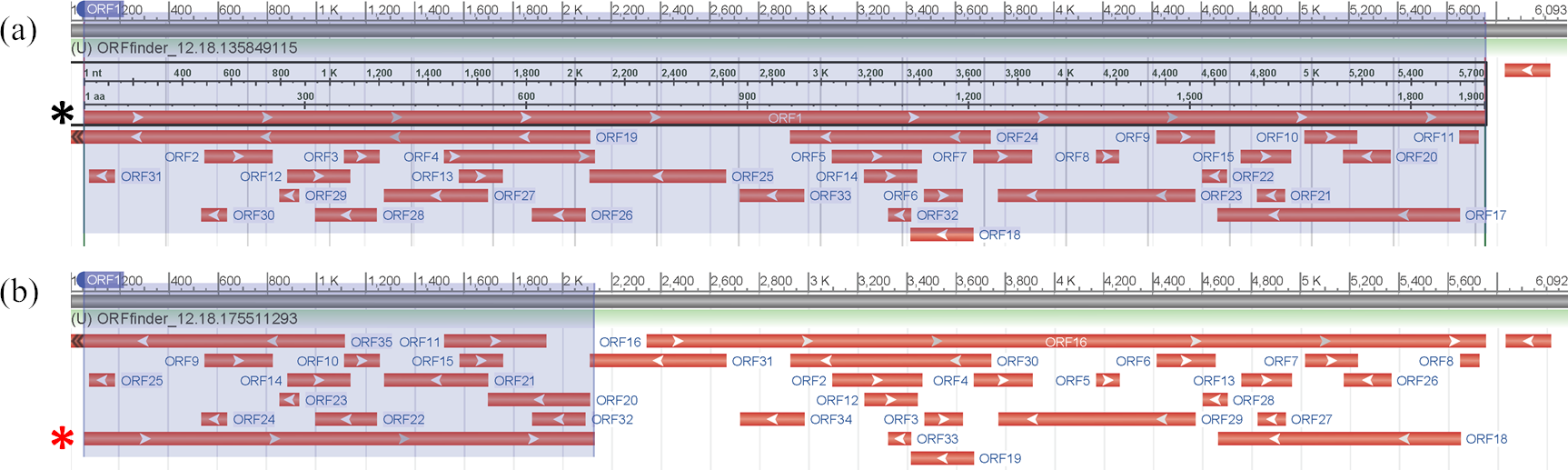

Supplement: Supplementary file 6 — Additional file 6: Figure S1. Comparison of predicted open reading frames (ORF) for OBSL1 mRNA (XM_027965226.1) and predicted mutant ovine mRNA for OBSL1 using NCBI ORF Finder [15] (accessed 18th December 2019, < https://www.ncbi.nlm.nih.gov/orffinder/>). (a) ORF1 (black *) represents the ORF that codes for OBSL1 (1899 amino acid residues). (b) ORF1 (red *) codes for a truncated and modified protein of 691 amino acid residues. [file 12863_2020_913_MOESM6_ESM.tif]

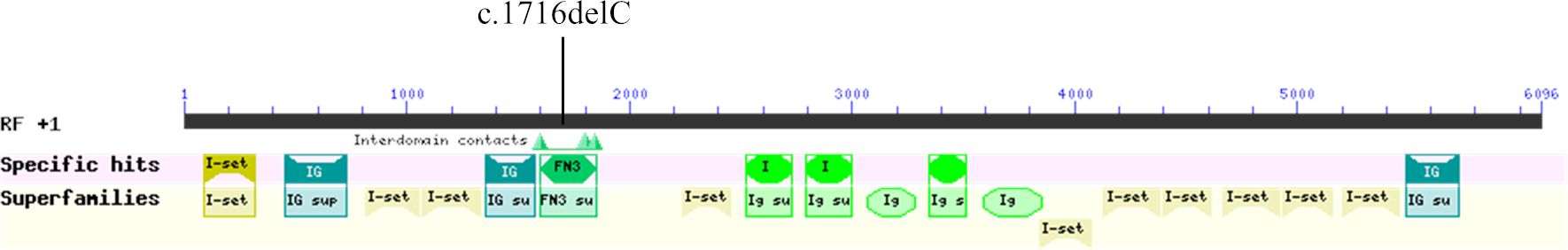

Supplement: Supplementary file 8 — Additional file 8: Figure S2. Wildtype OBSL1 protein showing conserved domains obtained from the NCBI Conserved Domains database [15, 54] (accessed 18th December 2019, <https://www.ncbi.nlm.nih.gov/Structure/cdd/wrpsb.cgi>). The location of the c.1716delC variant is indicated. The resulting modified protein p.(Val573Trpfs*119) is predicted to have a truncated fibronectin type 3 domain and is lacking four immunoglobulin domains. [file 12863_2020_913_MOESM8_ESM.tif]

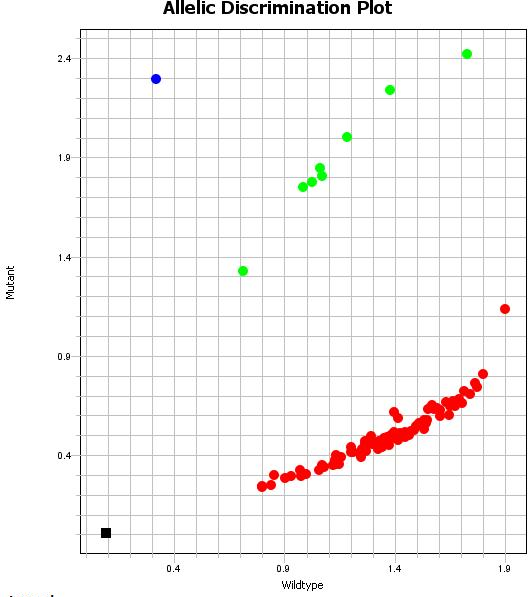

Supplement: Supplementary file 9 — Additional file 9: Figure S3. Allelic discrimination plot visualised using QuantStudio™ Real-Time PCR System version 1.3 (Applied Biosystems™) for a TaqMan genotyping assay used to discriminate the ENSOART00000022037.1:c.1716delC variant for homozygous wildtype (red dots), heterozygous (green dots), homozygous mutant (blue) individuals and a no DNA template control (black square). [file 12863_2020_913_MOESM9_ESM.tif]
