# Supplementary material for: Molecular basis of a new ovine model for human 3M syndrome-2
Source: BMC Genet. 2020 Sep 15;21:106. doi: 10.1186/s12863-020-00913-8 (PMC7493961; doi:10.1186/s12863-020-00913-8)
Supplement: Supplementary file 7 — Additional file 7. Ovine OBSL1 isoform X1 protein sequences (XP_027821027) for wildtype (SheepWT) and mutant (SheepMT) sheep. The predicted mutant c.1716delC (p.(Val573Trpfs*119)) altered amino acid sequence is highlighted in red. [file 12863_2020_913_MOESM7_ESM.pdf]

Additional file 7 Ovine OBSL1 isoform X1 protein sequences (XP_027821027) for wildtype (SheepWT) and mutant (SheepMT) sheep. The predicted mutant c.1716delC (p.(Val573Trpfs*119)) altered amino acid sequence is highlighted in red.

>SheepWT

MKAGSGDQGSPPCFLRFPRPVRVVSGAEAELKCVVLGEPPPIVVWEKGGQ
QLAASDRLSFPVDGAEHCLLLSGALPTDAGVYVCRARNSAGEAYAAAAVT
VLEPPAPEPEPQLAERPLPPPGAGEGAPVFLTGPRSQWVLRGAEVVLECQ
VGGLPAPTLYWEKDGMALDEVWDSSHFSLEPGRAGAGASLALRILAARLP
DSGVYVCHARNAHGHARAGALLQVQQPPESPPEDPDEAPTPVVEPLKCAP
KTFWVNEGKHAKFRCYVMGKPEPEIEWHWEGRPLLPDRRRLMYRDRDGGF
VLKVLYCQAKDRGLYVCAARNSAGQTLSAVQLHVKEPRLRFSRPLQDVEG
REHGIAVLECKVPNSRIPTAWFREDQRLLPCRKYEQIEEGTVRRLIIHRL
KADDDGVYLCEMRGRVRTVANVTVKGPILKRLPRKLDVFEGENAVLLVET
REAGVEGRWSRDGEDLPATCQSSSGHMHALVLPGVTREDAGEVTFSLGNS
RTTTLLRVKCIKHSPPGPPVLAEMFKGHRNTVLLTWKPPDPTPETAFIYR
LERQEVGSEDWVQCFSIEKAGAVEVPGDCVPTEGDYRFRVCTVSEHGRSP
HVVFHGSAHLVPTARLVAGLEEVQVYDGEDAVFSLDLSTVIQGTWFLNGE
ELKSNEPEGQVGPGPLRYRVEQRGLQHRLILQAVRHQDSGALIGFSCPGV
QDSAALTIQESPVHILSPQDKVSLTFTTSDRVVLTCELSRVDFPASWYKD
GQQVEESESLVVKMDGRKHRLILPEAQVQDSGEFECRTEGVSAFFSVTVQ
DPPVHIVAPREHVFVHAITSECVMLTCEVDREDAPVHWFKDGQEVEESDF
VLLESEGPHHRLVLPSAQPSIGGEFQCVAGDERAYFTVTITDVSSWIVYP
SGKVYVAAVRLERVVLTCELCRPWAEVRWTKDGEEVVESPTLLLQKEDTV
RRLVLPAVQLEDSGEYLCEIDDESASFTVTVTEPPVRILYPRDEVTLVAV
SLECVVLMCELSREDAPVRWYKDGLEVEESEALVLESDGPRRRLVLPAAQ
PQDGGEFVCDAGDDSAFFTVTVTAPPERIVHPAARSLDLQFRAPGRVELR
CEVAPAGSQVRWYKDGLEVEASEALQLGAEGPTRTLTLPHAQPEDAGEYV
CETRDEAVTFNVSLAEPPVQFLAPEAAPGPLCVAPGEPVVLSCELSRAGA
LVFWSHNGKPVQTGEGLELRAEGPRRVLCIRAADLAHAGLYTCQCGAAPG

APSLSFTVQVAEPPVRVVAPEAAQTRVRSTPGGDLELAVRLSGPGGPVRW

YKDGERLASQGRVQLEQDGARQVLRVRGARSRDAGEYLCDTPQDSRIFLV

SVEEPPLVKLVSELTPLTVHEGDDATFRCEVSPPDADITWLRNGVVITPG

PQLETTQNGSSRTLTVRSCRLEDAGTVTARAGGTSTSARLHVRETELLFL

RRLQDVRAEEGQDVCLEVETGRVGAAGAVRWVRGGAPLPPDSRLSTAQDG

HVFRLFIHSVVLADQGTYGCESHHDRTLARLSVRPKQLRVLRPLEDVTII

EGGNATFQLELSQEGVTGEWARGGVRLQPGPKCQIQAEGPTHHLVLSGLG

LADSGCISFTADTLRCAARLTVREAPVTIVRGLQDLEVTEGDTATFECAL

SQALADVTWEKDGQPLTPSARLRLQALGTRRLLQLRRCSPLDAGTYSCVV

GMARTGPVHLVVRERKVSVLSELRSVSAREGDGATFECTVSEVETAGSWE

LGGRPLRPGGRVRIRQEGKKHILVLSELRAEDAGEVRFQAGPAQSVAQLE

VEALPLQMRRRPPREKTVLVGRRAVLEVTVSRPGGQVCWLREGAELCPGD

KYQLRSHGPTHSLVIHDVRPEDQGTYCCRAGQDSAYTRLLVEGDAPLST

>SheepMT

MKAGSGDQGSPPCFLRFPRPVRVVSGAEAELKCVVLGEPPPIVVWEKGGQ

QLAASDRLSFPVDGAEHCLLLSGALPTDAGVYVCRARNSAGEAYAAAAVT

VLEPPAPEPEPQLAERPLPPPGAGEGAPVFLTGPRSQWVLRGAEVVLECQ

VGGLPAPTLYWEKDGMALDEVWDSSHFSLEPGRAGAGASLALRILAARLP

DSGVYVCHARNAHGHARAGALLQVQQPPESPPEDPDEAPTPVVEPLKCAP

KTFWVNEGKHAKFRCYVMGKPEPEIEWHWEGRPLLPDRRRLMYRDRDGGF

VLKVLYCQAKDRGLYVCAARNSAGQTLSAVQLHVKEPRLRFSRPLQDVEG

REHGIAVLECKVPNSRIPTAWFREDQRLLPCRKYEQIEEGTVRRLIIHRL

KADDDGVYLCEMRGRVRTVANVTVKGPILKRLPRKLDVFEGENAVLLVET

REAGVEGRWSRDGEDLPATCQSSSGHMHALVLPGVTREDAGEVTFSLGNS

RTTTLLRVKCIKHSPPGPPVLAEMFKGHRNTVLLTWKPPDPTPETAFIYR

LERQEVGSEDWVQCFSIEKAGAWRCPGTACLPKATTASESALSANTAAAP

TWCSTGLLTSCPQLAWWLVWRRYRCMMGKTPSSPWISPPSSRAPGSLTGR

SSRVTSQRARWGPGPCGTGWNSVACSTGSSCRPSGIRTAGP
